# PROTOCOL OF A RANDOMIZED, PARALLEL, DOUBLE-BLIND, PLACEBO-CONTROLLED PILOT TRIAL OF A KETONE ESTER IN AGING

**DOI:** 10.1093/geroni/igad104.2246

**Published:** 2023-12-21

**Authors:** Brianna Stubbs, Sawyer Peralta, Stephanie Roa Diaz, Gabriela Alvarez Azanedo, Laura Alexander, Wendie Silverman-Martin, James Johnson, John Newman

**Affiliations:** Buck Institute, Novato, California, United States; Buck Institute, Novato, California, United States; Buck Institute, Novato, California, United States; Buck Institute, Novato, California, United States; Buck Institute, Novato, California, United States; Buck Institute, Novato, California, United States; NA, Greenbrae, California, United States; Buck Institute, Novato, California, United States

## Abstract

**Background:**

Ketone bodies function as an energy and signaling metabolite and are hypothesized to interact with several geroscience pathways. Ketone esters (KE) are a nutritional tool that induce ketosis without other dietary changes; no studies have addressed KE in an aging population.

**Objectives:**

The primary objective of this randomized, placebo-controlled, double-blinded, parallel-group, pilot study is to determine the tolerability of 12-weeks of KE ingestion in older adults (>65y). Secondary outcomes include safety and acute ketone kinetics. Exploratory outcomes include physical function, cognitive function, quality of life, general and inflammatory aging biomarkers and gut microbiome.

**Methods:**

Community dwelling older adults who are independent in activities of daily living, and with no unstable acute medical conditions (n=30) will be recruited. The study intervention is a KE (n=15) or a placebo (PLA). Firstly, ketone kinetics after 12.5 or 25g of KE consumption will be assessed over four hours. Secondly, after collection of baseline safety, functional and biological measurements, participants will be randomly allocated to consume KE or PLA daily for 12-weeks. A questionnaire will assess tolerance daily for 2-weeks, and then via recall at 2-week intervals. Safety assessments will be repeated at week 4. All measures will be repeated at week 12.

**Conclusion:**

This study will provide data demonstrating the feasibility, tolerance, and safety of 12-weeks of KE consumption in older adults as well as exploratory data across a range of geroscience-related endpoints. This will facilitate our long-term goal: use of KE to study geroscience mechanisms and clinical outcomes relevant to frailty.

